# Sorting Gold and Sand (Silica) Using Atomic Force Microscope-Based Dielectrophoresis

**DOI:** 10.1007/s40820-021-00760-x

**Published:** 2021-12-04

**Authors:** Chungman Kim, Sunghoon Hong, Dongha Shin, Sangmin An, Xingcai Zhang, Wonho Jhe

**Affiliations:** 1 Department of Physics and Astronomy, Seoul National University, Seoul, 08826 Republic of Korea; 2 John A. Paulson School of Engineering and Applied Sciences, Harvard University, Cambridge, MA 02138 United States; 3 Department of Chemistry and Chemical Engineering, Inha University, Incheon, 22212 Republic of Korea; 4 Department of Physics, Institute of Photonics and Information Technology, Jeonbuk National University, Jeonju, 54896 Korea; 5 School of Engineering, Massachusetts Institute of Technology, Cambridge, MA 02139 United States

**Keywords:** Dielectrophoresis-empowered Pipette/AFM platform, On-demand materials sorting, Additive 3D printing, Multimaterial nano-patterning, Nanopipette-based atomic force microscope

## Abstract

Additive manufacturing–also known as 3D printing–has attracted much attention in recent years as a powerful method for the simple and versatile fabrication of complicated three-dimensional structures. However, the current technology still exhibits a limitation in realizing the selective deposition and sorting of various materials contained in the same reservoir, which can contribute significantly to additive printing or manufacturing by enabling simultaneous sorting and deposition of different substances through a single nozzle. Here, we propose a dielectrophoresis (DEP)-based material-selective deposition and sorting technique using a pipette-based quartz tuning fork (QTF)-atomic force microscope (AFM) platform DEPQA and demonstrate multi-material sorting through a single nozzle in ambient conditions. We used Au and silica nanoparticles for sorting and obtained 95% accuracy for spatial separation, which confirmed the surface-enhanced Raman spectroscopy (SERS). To validate the scheme, we also performed a simulation for the system and found qualitative agreement with the experimental results. The method that combines DEP, pipette-based AFM, and SERS may widely expand the unique capabilities of 3D printing and nano-micro patterning for multi-material patterning, materials sorting, and diverse advanced applications.

**Supplementary Information:**

The online version contains supplementary material available at 10.1007/s40820-021-00760-x.

## Highlights

- The dielectrophoresis-based platform combined with micropipette-based atomic force microscope is demonstrated for material- and position-selective deposition.
- The feasibility of on-demand sorting and printing using multi-materials in the single reservoir through a (sub)-microscale nozzle in the ambient condition is presented.

**Supplementary Information:**

The online version contains supplementary material available at 10.1007/s40820-021-00760-x.

## Introduction

Materials patterning and materials sorting are fundamentally important for diverse materials applications; they have been empowered by modern materials technologies such as additive manufacturing and microfluidics [1–9]. Additive manufacturing–also known as 3D printing–has attracted much attention in recent years as a powerful fabrication method for complicated structures. Due to its easy and simple process, it has been applied in various research and industrial fields and even in electronics [10–12]. The methods generally used in the 3D printing technique include fused deposition modeling [13], stereolithography [14], and selective laser sintering [15]. However, such methods have the limitation that they only use a single source of material in the nozzle during the printing process, which confines their applications in areas such as the on-site fabrication of hetero-materials and separate patterning with different species of printing materials.

Dielectrophoresis (DEP), a phenomenon where a force is exerted on a dielectric material in a non-uniform electric field in a liquid medium, can be applied as a materials sorting method [16, 17]. Many studies of DEP have focused on working in a microfluidic device for various materials such as cells [18, 19], bacteria [20], nanoparticles [21], carbon nanotubes [22], and DNA [23]. These studies are commonly performed in aqueous environments, so it is difficult to directly apply to the ambient conditions in printing technology.

Here, we introduce an advanced selective deposition and sorting technique with DEP-empowered Pipette/QTF-AFM (DEPQA), demonstrating multi-material patterning and materials sorting through a single nozzle in the ambient condition. The pulled pipette-combined quartz tuning fork-atomic force microscope (Pipette/QTF-AFM) demonstrating in free-standing liquid meniscus channel allows efficient low-volume liquid delivery and deposition in ambient conditions. DEPQA establishes the method for making micro/nanoscale water meniscus liquid channels for accurate and efficient printing, which is based on the precise feedback control of the distance between the tip and substrate without breaking the tip apex. Moreover, the system has the capability of microscopy imaging and in situ analysis of the mechanical responses of the micro/nanoscale liquid channel using QTF signal calculations. We emphasize that this system allows not only the delivery of low-volume liquid but also the selective deposition of different species of materials: Au and silica nanoparticles contained in a single solute. First, we simulated the DEP force profiles between the apex of the pulled pipette and an Au-coated glass substrate. The selective deposition of Au and silica nanoparticles was demonstrated experimentally with the DEPQA. Moreover, we discuss the electrothermal effect of DEP including applications such as surface-enhanced Raman spectroscopy (SERS) of the selectively patterned materials and the real-time motion of the ejected nanoparticles by monitoring the amplitude and phase responses of the QTF sensor. We believe that the results provide a different approach for multi-material patterning and materials sorting and a wider viewpoint for additive manufacturing and expand the applications of DEP-based research and industry.

## Experimental Section

**Fig. 1:**
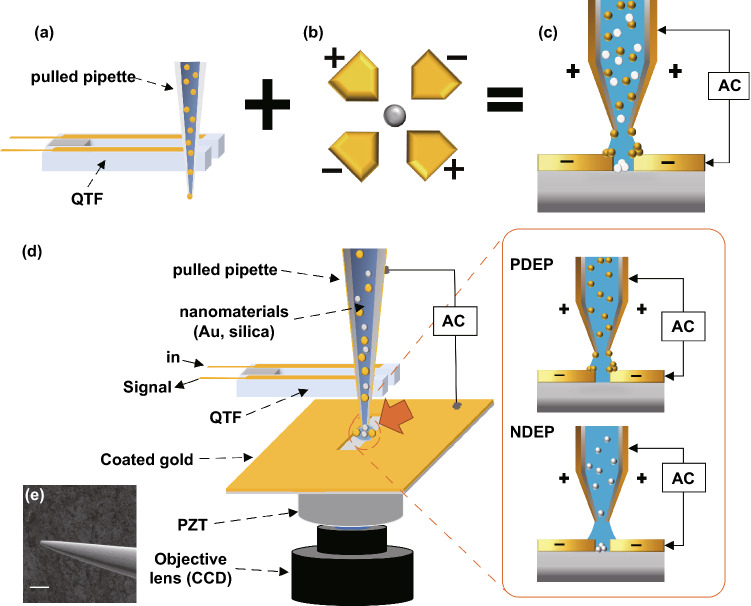
Schematic diagram of the experiment. **a** QTF-AFM with a pulled pipette system. It has strength in efficient low-volume liquid delivery. **b** Quadrupole DEP trap. If the Clausius–Mossotti factor is below zero, solutes receive a negative-DEP force and go to the center of the electrodes. **c** We propose the DEP-based material-selective deposition technique using the pipette-AFM system. The pipette-AFM delivers the low-volume solution to the desired position and DEP forces deposited nanoparticles on the edge or middle position of electrodes. Yellow particles show the positive-DEP effect and white particles show the negative-DEP effect. **d** Detailed schematic diagram of the experimental setup. XY positioning of the pipette on the scratched gap is enacted while watching the optical microscope image of CCD in real-time, and the Z positioning (approach and retract) of the pipette is enacted under QTF-AFM feedback. An AC voltage is applied between the coated Au layer on the glass substrate and the surfaces of the Au-coated pulled pipette. **e** Detailed SEM image of the coated pulled pipette, 10 µm scale bar

Figure 1 presents the schematic of the DEPQA system. Figure 1a-c show the pulled pipette-combined QTF-AFM, quadrupole negative-DEP trap, and their combined structures, respectively, allowing the selective deposition without a microfluidic device or the aqueous environment. Figure 1d shows a more detailed structure. Exploiting the pipette-combined QTF-AFM allows nano-to-sub-nanometer scale distance control between the orifice and substrate possible by sensitively monitoring the amplitude and phase variation of the AFM tip with very high quality factor [24]. In addition, QTF, as a highly sensitive force sensor, can measure the mechanical properties of the micro/nanoscale liquid channel [25, 26]. Therefore, by oscillating the QTF at a resonance frequency near 32 kHz with sub-nanometer amplitude, we employ this approach, stop, and retract processes while measuring the signal of QTF in real-time to check the water meniscus (channel) formation and monitor what takes place. The apex of the pipette was positioned at the center of the gap with guidance of an optical microscope, establishing a quadrupole electrode shape that allows materials to be deposited at different positions depending on the exerted DEP force. Figure 1e shows the scanning electron microscope (SEM) image of the tip aperture which has microscopic diameter.

To fabricate the pipette (aperture diameter ~2 µm), a quartz tube (O.D.: 1 mm, ID: 0.7 mm) was stretched by a mechanical puller (P-2000, Sutter Instrument Co.) and then Cr (5 nm, K-575X, Emitech) and Au (30 nm, SPT-20, COXEM) were consecutively sputter-coated on both sides of the pipette. On the other hand, the underlying substrate was prepared using the same coating method on cover glass (No. 23 550 32, DURAN GROUP), which was then scratched by a buckled pipet to fabricate the metallic gap [27]. By controlling the buckled depth, we could make a ~2 µm gap that is very similar to the pipet diameter. The gap width was kept invariant by translating the tip fastly ($\ge$ 10 μm s^-1^). We used an Au nanoparticle solution (40 nm diameter, OD 1, stabilized suspension in citrate buffer, Sigma Aldrich) after twice removing surfactants. The solution was centrifuged for 30 min at 13,500 rpm and redispersed in deionized (DI) water. Silica nanoparticle solutions (JSD0051, nanoComposix Inc.) were diluted 10 times to prevent rapid aggregation. Au and silica nanoparticle solutions were mixed together while keeping each concentration the same. To prevent the clogging phenomenon during pipetting, stabilizing polymer (polyvinylpyrrolidone) was added into the mixed nanoparticle solution.

## Results and Discussion

### DEP Simulation

Equations (1) and (2) describe the basic theory of the experiment: DEP force equation of a uniform sphere (diameter a) in a medium of complex permittivity ($\tilde{\varepsilon }_{{\text{m}}}$) [28, 29]. In this equation, the Clausius–Mossotti factor ($f_{{{\text{CM}}}} )$ determines the strength and the direction of the DEP force. If the real part of $f_{{{\text{CM}}}}$ is positive, particles are attracted to the position where $\left| {\vec{E}} \right|^{2}$ is maximized; positive DEP (PDEP, Fig. 1d). If the real part of $f_{{{\text{CM}}}}$ is negative, particles experience repulsive force from $\left| {\vec{E}} \right|^{2}$ maximum; negative DEP (NDEP, Fig. 1d). $f_{{{\text{CM}}}}$ depends on the difference between the complex permittivity of particles ($\tilde{\varepsilon }_{{\text{p}}}$) and medium ($\tilde{\varepsilon }_{{\text{m}}}$). Therefore, the motion under DEP forces depends on diameter, frequency (${\upomega })$, and characteristic parameters of solutes and medium ($\varepsilon_{{\text{m/p}}} { }$ bulk permittivity, $\sigma_{{\text{m/p}}}$ conductivity, $\sigma_{{{\text{bulk}}}}$ bulk conductivity, and $K_{{\text{s}}}$ surface capacitance).

1 $$\langle F_{{_{{{\text{DEP}}}} }} \rangle = \pi \varepsilon_{{\text{m}}} a^{3} {\text{Re}} \left( {f_{{{\text{CM}}}} } \right)\nabla \left| {\vec{E}} \right|^{2}$$

2 $$f_{{{\text{cm}}}} = \frac{{\tilde{\varepsilon }_{{\text{p}}} - \tilde{\varepsilon }_{{\text{m}}} }}{{\tilde{\varepsilon }_{{\text{p}}} + 2\tilde{\varepsilon }_{{\text{m}}} }}\,\,{\text{where}}\,\,\tilde{\varepsilon }_{{{\text{m/p}}}} = \varepsilon _{{{\text{m/p}}}} - i\frac{{\sigma _{{{\text{m/p}}}} }}{\omega }{\text{ }}\,{\text{and }}\sigma _{{{\text{m/p}}}} = \sigma _{{{\text{bulk}}}} + 2\frac{{K_{{\text{s}}} }}{a}$$

Simulation results indicate the various phase statuses of the electrodes, as shown in Fig. S1, to define the optimal geometry of the tip and sample parts. From Eq. (1), the solutes are attracted to the high $\left| {{\vec{\text{E}}}} \right|^{2}$ region in the PDEP case and to the low region in the NDEP case. All cases make $\left| {{\vec{\text{E}}}} \right|^{2}$ maximum at the edges of the electrodes. However, just Fig. S1a case makes $\left| {{\vec{\text{E}}}} \right|^{2}$ a minimum at the center of electrodes. Therefore, we use the phase status of Fig. S1a.

**Fig. 2:**
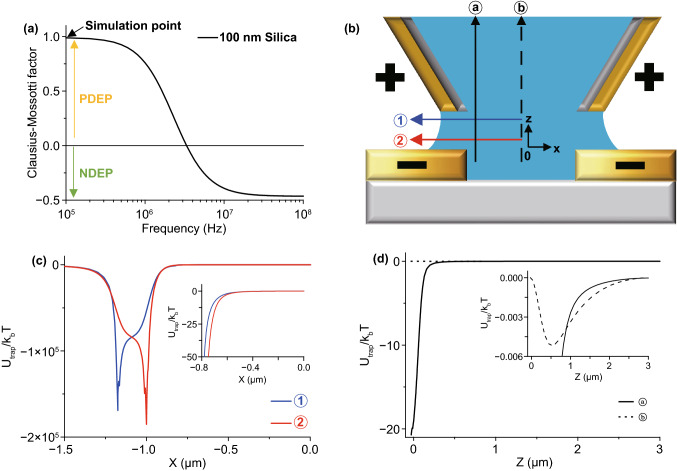
**a** An example graph of Clausius–Mossotti factor ($f_{{{\text{CM}}}}$) of 100 nm diameter silica nanoparticles. The sign of $f_{{{\text{CM}}}}$ determines PDEP or NDEP. The critical frequency of the sign is almost 3 MHz in this case. **b** Schematic diagram of the experiment with arrows for the X- and Z-directions. The phase of electrodes is determined by the simulation in Fig. S1. **c** Simulation graph of PDEP ($f_{{{\text{CM}}}}$ = 1.0) trap energy vs. Brownian motion along the X-axis. $V_{{{\text{peak}}}}$ = 1.0 V, 15 MHz frequency of AC voltage, 100 nm diameter of the particle, 2.0 µm gap size, and 2.35 µm outer diameter of the pipette with a 0.7 $R_{{{\text{inner}}}} /R_{{{\text{outer}}}}$ ratio. The trap energy rapidly increases around the edge of electrodes near the outer diameter of the pipette at ① (±1.175 µm) and substrate at ② (±1.0 µm), so the particles affected by the PDEP force are supposed to move to the edge of the electrodes. The inset image shows an expanded graph near the center; it shows the same direction for the PDEP force. The NDEP case shown in Fig. S3 and the particles affected by NDEP gather around the center near zero. **d** Simulation graph of the DEP trap energy vs. Brownian motion along the Z-axis with the same conditions as **c**. The particles affected by PDEP force are supposed to move downward at ⓐ but the particles affected by NDEP go upward. However, in the inset graph, the Brownian motion energy is much bigger than the z-direction DEP trap energy at ⓑ, so particles are not affected by the DEP force in the Z-direction except near ⓐ

Figure 2 shows the simulation results of pre-examination to determine the experimental parameter. Figure 2a shows the calculation result of the frequency and real part of $f_{{{\text{CM}}}}$ of the 100 nm diameter silica nanoparticle. The frequency of a zero-point of $f_{{{\text{CM}}}}$ near 3 MHz is the critical frequency $(\omega_{{\text{c}}} )$, so the particles are attracted by PDEP below $\omega_{{\text{c}}}$ and NDEP above $\omega_{{\text{c}}}$. Figure 2c, d show the trap energy from the PDEP force vs. the Brownian motion energy ($= k_{{\text{B}}} T$) in the experimental scheme with the directions in Fig. 2b. The trap energy is calculated as $- \pi \varepsilon_{{\text{m}}} a^{3} \left| {\vec{E}} \right|^{2}$ from Eq. (1) where $Re\left( {f_{{{\text{CM}}}} } \right) = 1$; the PDEP condition. Minimum points appear at the outer edges of the pipette at ① (blue arrow in Fig. 2b) and the edges of the electrodes on substrates at ② (red arrow in Fig. 2b) in Fig. 2c. We expect that particles in the PDEP cases are attracted to the edges of electrodes. In addition, we calculated the NDEP case as $- \frac{1}{2}\pi \varepsilon_{{\text{m}}} a^{3} \left| {\vec{E}} \right|^{2}$ from Eq. (1) with the NDEP condition, $Re\left( {f_{{{\text{CM}}}} } \right) = - \frac{1}{2}$, as shown in Fig. S2. In the NDEP case, the maximum points appear at edges of electrodes, so particles experience a repulsive force from electrodes and aggregate at the center that is a minimum point in Fig. S2a. We simulate the trap energy of the DEP force with the *z*-direction in Fig. 2d. The trap energy is 20 times bigger than the Brownian motion ($k_{{\text{B}}} T$) near the substrate at ⓐ. Thus, the particles in the pipette near-wall would descend to the maximum point in the PDEP case and particles ascend in the NDEP case as shown in Fig S2b. However, particles in the pipette except ⓐ would be not affected by the *z*-direction DEP force more than Brownian motion from the ⓑ result in Fig. 2d. As a result of the *x*- and *z*-direction results, the PDEP force is directed toward the edges of the electrodes and the NDEP force is directed toward the center.

### DEPQA Demonstration

DEP confirming of nanoparticles undergo the processes in Fig. S3. When the z-position of the pipette is fixed near 750 nm on the gap using the feedback of the DEPQA system, alternating current (AC) voltage is applied between surface electrodes of pipette and substrate using a function generator (Model #33519B, Keysight Technologies). Just after applying, the pipette is approached until a water meniscus forms (*z* = ~80 nm) and is fixed at that position for either 30 or 60 s. We define the formation point of a liquid channel between the tip apex and the surface by monitoring the sudden change of amplitude and phase signals of the QTF sensor. After all processes are finished, the pipette is retracted almost 10 µm instantaneously to break the liquid channel rapidly. Note that if we retract the pipette slowly, a wire of aggregated nanoparticles and surfactants forms between the tip apex and the substrate by a streaking process such as a 3D printing schematic.

#### Motion of the Ejected Nanoparticles

During liquid delivery and materials deposition, we found two different behaviors of particles by monitoring the amplitude signal of the QTF sensor. In the first one, the amplitude signals are oscillation signals with a period of a few seconds until 30 seconds as shown in Fig. S4. We assume that this oscillation is the beating pattern of incompletely compensated original stray capacitance signal and QTF output signal [30]. The period of the pattern is almost 5 seconds in Fig. S4a, so the beating frequency (${\text{f}}_{{{\text{beat}}}} = \Delta {\text{f}} = {\text{f}} - {\text{f}}_{0}$) is ~0.2 Hz. The equation of the frequency shift of QTF is as Eq. (3) [31, 32]:

3 $$\frac{1}{{\omega^{2} }} - \frac{1}{{\omega_{0}^{2} }} = \frac{\Delta m}{k}$$

This equation can be adjusted as Eq. (4):

4 $$\Delta m \cong 2{\text{k}}\frac{\Delta \omega }{{\omega_{0}^{3} }}$$

From the last equation, $\Delta m$ is calculated as $2.819 \times 10^{ - 13} { }$ kg (k ≈ 1,000 N m^-1^, $f_{0}$ ≈ 33 kHz approximately), which indicates that 54,490 counts of Au nanoparticles are gathered and clustered at the edge of the pipette by PDEP. With this oscillation information (beating pattern), we may roughly estimate the number of particles enriched at the edge of the pipette oscillation. Second, the oscillating signals after 30 s show disappearance in Fig. S4a, the Au case, and the sharp amplitude decrement of QTF sensor indicates the solidification behavior of the liquid nanochannel observed in Fig. S4b-d. This is because the Au particles make a connection with the electrodes on the pipette apex and the substrate, making a large momentary current that results in resistive loss and the rapid evaporation of liquid in the nanochannel, resulting in the disconnection of the bridge. For other cases of the silica nanoparticles, the increasing local concentration may cause the aggregation of silica particles connecting the apex of the pipette and the substrate strongly, allowing the dramatic decrement of amplitude signal to near-zero value. Notice that we monitor particle dynamics through the pipette using the QTF signals, it will be interesting if there is a possibility to use such information to empirical choice of proper solutions for non-aggregation roles in the reservoir. In this regard, predicting the expected signal results for the metallic and dielectric particles may indicate whether the solute, solvent, and surfactants used are proper for solutions to achieve non-aggregation roles before obtaining the FESEM image of the results.

#### ${\varvec{f}}_{{{\text{CM}}}}$***of the Ejected Nanoparticles***

**Fig. 3:**
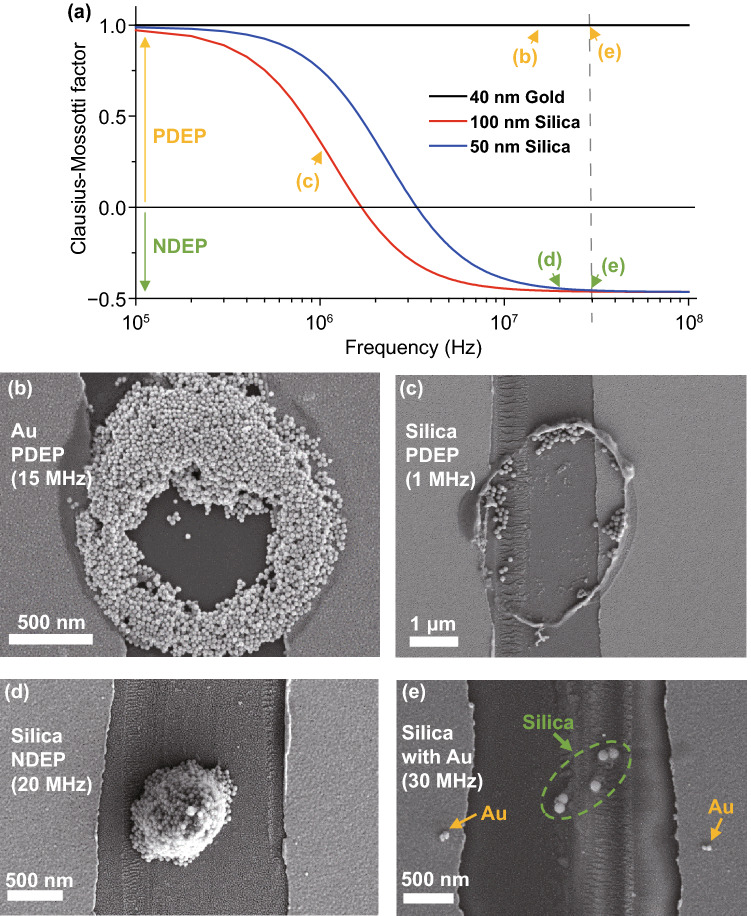
SEM images of PDEP and NDEP experiments. **a** Clausius–Mossotti factor (${\varvec{f}}_{{{\mathbf{CM}}}}$) of nanoparticles used for experiments. Several characteristics (${{\varvec{\upvarepsilon}}}_{{{\mathbf{m}}/{\mathbf{p}}}} , {{\varvec{\upsigma}}}_{{{\mathbf{m}}/{\mathbf{p}}}} , {{\varvec{\upsigma}}}_{{{\mathbf{bulk}}}} , {\mathbf{K}}_{{\mathbf{s}}}$) of Au and silica nanoparticles are from reference [29]. The X-axis is a log scale of the frequency (Hz). Signs of ${\varvec{f}}_{{{\mathbf{CM}}}}$ change at ~2 MHz (100 nm silica), ~4 MHz (50 nm silica), and ${\varvec{f}}_{{{\mathbf{CM}}}}$ of 40 nm Au always has a plus sign in the 0.1–100 MHz range. Arrows show the frequencies used, and colors indicate PDEP (yellow) or NDEP (green). **b** PDEP result of Au nanoparticles with 40 nm diameter Au nanoparticles, ~2 µm diameter pipette with ${\varvec{R}}_{{{\mathbf{inner}}}} /{\varvec{R}}_{{{\mathbf{outer}}}}$ ratio = 0.7, ${\varvec{V}}_{{{\mathbf{peak}}}} = \user2{ }$ 1.0 V, and 15 MHz frequency. Particles are attracted to surface electrodes of the substrate and the pipette. **c** PDEP result of silica nanoparticles with 100 nm diameter silica nanoparticles, ~ 2 µm diameter pipette, ${\varvec{V}}_{{{\mathbf{peak}}}} = \user2{ }$ 2.5 V and 1 MHz frequency. Particles show the same tendency as in **a**. The concentration and surfactants of nanoparticle solutions result in differences between **b and c. d** NDEP result of silica nanoparticles with 50 nm diameter silica nanoparticles, ~1.5 µm diameter pipette, ${\varvec{V}}_{{{\mathbf{peak}}}} = \user2{ }$ 2.5 V, and 20 MHz frequency. Particles move off to the surface electrodes of the substrate and pipette and gather around the center of the pipette. **e** Sorting result of the mixed solution with 100 nm diameter silica nanoparticles and 20 nm diameter Au nanoparticles, ${\varvec{V}}_{{{\mathbf{peak}}}} = \user2{ }$ 2.5 V, and 30 MHz frequency. Silica nanoparticles in the green circle show NDEP and Au nanoparticles next to the yellow arrows show PDEP

Figure 3a shows the simulation result of frequency and real part of the $f_{{{\text{CM}}}}$ of the nanoparticles used in the experiments. 40 nm Au nanoparticles are expected to show the motion of PDEP regardless of frequency at the experimental range (0.5–30 MHz). However, for the 50 and 100 nm silica nanoparticles, PDEP appears at a low frequency (~1 MHz) and NDEP appears at a high frequency (~100 MHz). With 100 nm diameter silica nanoparticles, near 3 MHz is a critical frequency between PDEP and NDEP. In addition, most of the metallic particles exhibit the same trends as the real part of $f_{{{\text{CM}}}}$ for Au nanoparticles, while most of the dielectric particles also show similar trends to silica nanoparticles with a different critical frequency. Therefore, with the proper choice of frequency applied at the electrodes, any well-dispersed materials other than the Au and silica pair could be selectively deposited in principle depending on the diameter and dielectric characteristic parameters of the solutes and medium. Note that best optimization of the sorting function for a particular pair of materials may require specific choice of surfactants as discussed in the next subsection, which is an interesting, practical issue to address, even with the help of machine learning for such search, which is the beyond the scope of the current work.

#### Proof-of-principal Demonstration of DEPQA Using Nanoparticles

Figure 3b–e are SEM images of the experimental results. Au nanoparticles are deposited in a circular shape in Fig. 3b, and it can be seen as an attraction to the edges of electrodes on the substrate and circular apex of the pipette; PDEP. The tendency of silica nanoparticles in Fig. 3c is similar to the Au case, which can also be seen as PDEP. The different concentrations and surfactants result in differences between the two cases. Silica nanoparticles in Fig. 3d gathered at the center of the pipette position, which shows that particles take repulsive force from electrodes; NDEP. Figure 3e shows the selectively deposited result of a mixed solution. Au nanoparticles (yellow arrows, small one) are deposited on both sides of electrodes that are the maximum points in Fig. 2c and silica nanoparticles (in the green circle) are deposited at the center of the electrodes. Therefore, we confirmed the feasibility of selective deposition using DEP expected with pre-examined simulation.

Note that to see the effective preconceived DEP effect, the ratio of the outer diameter of the pipette and the gap distance of the electrodes on the substrate should be similar as shown in the simulation graph in Fig. S5. If the ratio of the diameter to the gap distance is much bigger or smaller than 1, particles are not deposited at the expected position and are instead scattered like in Fig. S6. In addition, if there is no gap, particles are deposited randomly regardless of the type of solution like Fig. S7. These SEM images show that nanoparticles in Fig. 3 are definitely affected by the DEP force.

Clogging the orifice of the pipette and aggregation of the solutes before deposition is the main technical challenge in the experimental processes, and various methods have been attempted to address this practical issue. We have observed that UV/Ozone cleaning of the metal-coated pipette and using a quartz pipette rather than a borosilicate one actually slowed down the clogging. Moreover, we have found empirically that the more hydrophilic surface inside the pipette, like the above two methods, slows down the clogging problem although the exact mechanism is not clear. In addition, the resonance frequency of QTF used in the experiment (~32 kHz) is within the sonication band, and so we vibrated the pipette at an amplitude of a few micrometer between experiments. While this QTF-based sonication process also slows down the clogging, it does not prevent it entirely. If some chemical treatments that prevent entirely the clogging issue could be done before DEPQA experiment, it would be very helpful to make this work more attractive and practically important. For example, removal of the aggregates that clog the orifice, application of strong DC voltage (>100 V) between inside of the pipette and the substrate far from the deposition site could help address the issue, in addition to finding the best optimum surfactants common to the two types of nanoparticles.

The original solutions of nanoparticles even without any treatment prevent aggregation quite well. However, use of original solutions indicates that surfactants are also deposited at the same time and so just few particles are deposited because they could not be collected much near the orifice. Therefore, to enhance the sorting function, one has to do some proper treatment for the original solution, which is why aggregation is rather inevitable and limits the sorting capability. For example, when the removal of surfactants was executed for the Au nanoparticle solution, much more nanoparticles than the expected number in the water channel were deposited. On the other hand, centrifugation of the silica nanoparticle solution was rather challenging, so the surfactant concentration had to be reduced by dilution with deionized water. Even so, more silica nanoparticles than the expected number in the water channel were also deposited. In the mixed solution case of silica and Au nanoparticles, the silica nanoparticle solution was diluted down to the level similar to the concentration of the Au nanoparticle solution, while stabilizing polymer (polyvinylpyrrolidone) was added to the Au nanoparticle solution. Nonetheless, the well-dispersed conditions for each solution are different, and as a result, we could only obtain a few nanoparticles separated as shown in Fig. 3e. Further extensive and detailed search for the optimum suitable surfactants and solutions for the mixed case would certainly enhance the sorting function, which remains the theme of future research beyond our proof-of-principle demonstration of sorting function presented in this manuscript.

#### Electrothermal Effect

Figure S8 shows the potential obstacle to selective deposition; the electrothermal (ETE) effect [28]. Even the structural factors (the gap/pipette ratio and the correct positioning of the pipette on the gap) and the solution used are critical to the optimum conditions, heating near the electrodes also remains an important issue for selective deposition. From Eq. (1), the stronger electric field creates the stronger DEP force. However, the stronger electric field results presented in Fig S8a and b show not so well deposited at the selective area of PDEP (outside the pipette), because the high voltage makes the stronger DEP force and so a large amount of heat flow in the solution due to the ETE effect. The ETE effect can be expressed by Eqs. (5) and (6) [33]:

5 $$\begin{gathered} \langle \vec{F}_{{{\text{ETE}}}} \rangle_{{{\text{volume}}}} = 0.5\varepsilon \nabla T\vec{E}^{2} \Pi \left( w \right), \hfill \\ \Pi \left( w \right) = \left( {\frac{\alpha - \beta }{{1 + \left( {\omega \tau } \right)^{2} }} - \frac{\alpha }{2}} \right) , \hfill \\ \end{gathered}$$

6 $${\text{where }}\alpha = \left( {\frac{1}{\varepsilon }} \right)\left( {\frac{\nabla \varepsilon }{{\nabla T}}} \right) = - 0.4\% K^{ - 1} , \beta = \left( {\frac{1}{\sigma }} \right)\left( {\frac{\nabla \sigma }{{\nabla T}}} \right) = 2\% K^{ - 1}$$

In the equations, $\varepsilon$, $\sigma$, $\tau$, and $\omega$ are the permittivity and conductivity of the medium of the solution, the charge relaxation time $(\varepsilon /\sigma ),$ and frequency, respectively. ${\Pi }\left( {\text{w}} \right)$, the unit-less function of the frequency, determines the direction and magnitude of the ETE force. In the Au nanoparticle case, the solvent is a citrate buffer that has much higher conductivity than DI water even after twice removal of the surfactants. Since ${\Pi }\left( {\text{w}} \right)$ is negative ($\cong - 0.022$) in the experimental range of the frequency, the ETE force has the opposite direction with respect to the DEP force and the flow of the solution induced by ETE disturbs the PDEP-induced sorting. In addition, the $\nabla {\text{T}}$ term in (3) would increase depending on $\left| {{\vec{\text{E}}}} \right|$ because of Joule heating (${\text{k}}\nabla^{2} {\text{T}} + {\sigma E}^{2} = 0)$, so $\langle {\vec{\text{F}}}_{{_{{{\text{ETE}}}} }} \rangle$ increases more steeply depending on the electric field than $\langle \vec{F}_{{{\text{DEP}}}} \rangle$. Therefore, higher voltage amplitude has a lower accuracy for the spatial separation in Fig. S8a, b.

For quantitative analysis, the number rates of not accurately deposited particles are defined as the number of not accurately deposited particles/number of deposited particles times 100, and the accurate deposition area of PDEP is considered as toroidal, similar to the shape of a pipette hole (the outer and inner diameter ~ 2 and 1.4 $\mathrm{\mu m},$ respectively). The ETE effects in the experimental results appear as an increase of the rate parameters at 3 and 5 V in Fig. S8d (black squares). In addition, joule heating simulation was employed to calculate the ETE force in Eq. (5). The resistive losses at the liquid channel (an orange square in Fig. S8c) were calculated as 8.3×10^-12^, 7.5×10^-11^, and 2.1×10^-10^ W at 1 , 3, and 5 V, respectively, and assuming that the liquid channel has the similar thermal behavior with bulk liquid, $\nabla {\text{T}}$ could be obtained. Furthermore, $\frac{{\langle {\text{F}}_{{{\text{ETE}}}} \rangle }}{{\langle {\text{F}}_{{{\text{DEP}}}} \rangle }}$ could be estimated with $\left| {{\vec{\text{E}}}} \right|$ from the simulation results of Fig. 2c, d. The calculated $\frac{{\langle {\text{F}}_{{{\text{ETE}}}} \rangle }}{{\langle {\text{F}}_{{{\text{DEP}}}} \rangle }}$ are shown as a dashed line in Fig. S8d. We can assume that the rate of not accurately deposited particles has strong correlation with $\frac{{\langle {\text{F}}_{{{\text{ETE}}}} \rangle }}{{\langle {\text{F}}_{{{\text{DEP}}}} \rangle }}$ since the graph shows similar trends between black squares and dashed lines. In other words, the main reason for the lowered accuracy of spatial deposition at a higher voltage is associated with the ETE effect and it is very important to use the optimum value of the AC voltage that creates sufficient DEP force and a low rate of $\frac{{\langle {\text{F}}_{{{\text{ETE}}}} \rangle }}{{\langle {\text{F}}_{{{\text{DEP}}}} \rangle }}$. In addition, we considered the AC electro-osmosis effect occurring in the DEP experiment at the relatively high frequency (>0.5 MHz) used in the experiment; however, this makes the AC electro-osmosis effect negligible compared with ETE effect and DEP [34].

### Application for SERS

**Fig. 4:**
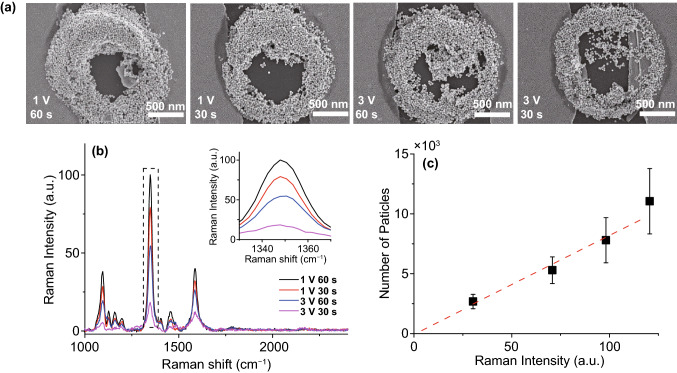
SERS results of various PDEP Au nano-aggregates samples. **a** SEM images of Au nano-aggregates deposited by PDEP; 500 nm scale bar. **b** SERS spectra of various Au nano-aggregates coated with 4-nitrobenzenethiol; the inset graph is the expanded spectra in the dotted line (nitro group). **c** Raman intensity and particle count graph. The ratio of Raman intensity of peaks in the inset graph of **b** is linearly well-fitted (red dashed line) with the ratio of the number of Au nanoparticles, which indicates that Au nanoparticles enhanced Raman signal. Therefore, the system can be used for the fabrication of the nano-micro SERS platform by the enrichment of nanoparticles such as in the coffee-ring effect

Figure 4 shows the SERS effect application of the selective deposition. We made sure that the site-selective samples had the SERS active property using PDEP combined Pipette/QTF-AFM. The 4-nitrobenzenethiol molecule was self-assembled on Au-coated substrates for the Raman measurement. The Raman signal of 4-nitrobenzenethiol (Fig. 4b) was only detected at the site of the deposited area of Au nanoparticles (Fig. 4a). In addition, in Fig. 4c, the Raman intensity vs. the number of particles is linearly well fitted, which indicates that the Au nanoparticles of the results demonstrate the SERS effect. Nanoparticles are widely used to make SERS samples using the coffee-ring effect [35], or other complicated processes [36]. Using the DEPQA system, one can make the SERS platform of enriched nanoparticles efficiently and control the SERS activity with the amount of solution in a very small area. Fig 4a shows enriched nanoparticles like the coffee-ring effect and much more particles are enriched on a substrate compared to the concentration. For example, the number of nanoparticles at 1 V, 60 s in Fig. 4a is the quantity in ~111 pL solution of the original concentration, and 111 pL is the amount of the pipette end to ~200 µm height (the half-angle of the pipette is assumed as 5°), which is 44,200 times larger amount than the solutes in the water channel. Therefore, it is shown that the solutes are enriched in our experimental scheme and they can be used for the SERS-active substrates.

## Conclusion

The selective deposition of various materials in one nozzle can provide a wide capability of additive manufacturing for greater diversity of materials and simplified processes. Au nanoparticles and silica nanoparticles showed the DEP effect as expected from the simulation and were selectively deposited spatially. Therefore, we demonstrated selective deposition of the Au and silica nanoparticles at the calculated frequency. However, the sorting capability was limited by the clogging and aggregations issues, and we discussed about these technical challenges, various methods to address, and the resulting associated effects. The electrothermal effect that is a potential obstacle for this system was analyzed and the appropriate voltage range was found. In addition, we confirmed monitoring particle dynamics roughly from QTF signals and the SERS effect of our experimental result. With this scheme, one can perform more diverse applications in the field of the additive manufacturing. We confirmed the feasibility of selective deposition using the DEP of Au and silica nanoparticles with a single pipette with the guidance of QTF-AFM in ambient conditions along with simulated parameters. In addition, the dynamics of the nanoparticles confined in a water channel were monitored roughly in real-time and the SERS effect was confirmed. The principal demonstration was implemented with our home-made system and a significant effect could exist depending on the operator. However, by updating the system more user-friendly, DEPQA would be a powerful tool for nanoscale sorting and fabricating three-dimensional structures such as the 3D nanowires with metal-bound and dielectric cores or other complicated structures in ambient conditions with suitable solutions, which may be useful for additive manufacturing feature of 3D printing. Besides, the demonstration may widely expand the capability of DEP, which can be applied to SERS, nano-micro selective patterning, materials sorting, biomedical engineering, and many other applications.

## Supplementary Information

Below is the link to the electronic supplementary material.

Supplementary file1 (PDF 702 kb)
