# Supplementary material for: Sorting Gold and Sand (Silica) Using Atomic Force Microscope-Based Dielectrophoresis
Source: Nanomicro Lett. 2021 Dec 4;14:13. doi: 10.1007/s40820-021-00760-x (PMC8643387; doi:10.1007/s40820-021-00760-x)
Supplement: Supplementary file 1 — Supplementary file1 (PDF 702 kb) [file 40820_2021_760_MOESM1_ESM.pdf]

Supporting Information for

Sorting Gold and Sand (Silica) Using Atomic Force Microscope-Based Dielectrophoresis

Chungman Kim[1, 2], Sunghoon Hong[1], Dongha Shin[1, 3], Sangmin An[1, 4], Xingcai Zhang[2, 5, *] and Wonho Jhe[1, 2, *]

[1]Department of Physics & Astronomy, Seoul National University, Seoul 08826, Republic of Korea

[2]John A. Paulson School of Engineering and Applied Sciences, Harvard University, Cambridge, MA, 02138, United States

[3]Department of Chemistry and Chemical Engineering, Inha University, Incheon 22212, Republic of Korea

[4]Department of Physics, Institute of Photonics and Information Technology, Jeonbuk National University, Jeonju 54896, Korea

[5]School of Engineering, Massachusetts Institute of Technology, Cambridge, MA, 02139, United States

***Corresponding author. E-mail: whjhe@snu.ac.kr (Wonho Jhe), xingcai@mit.edu (Xingcai Zhang)

Supplementary Figures

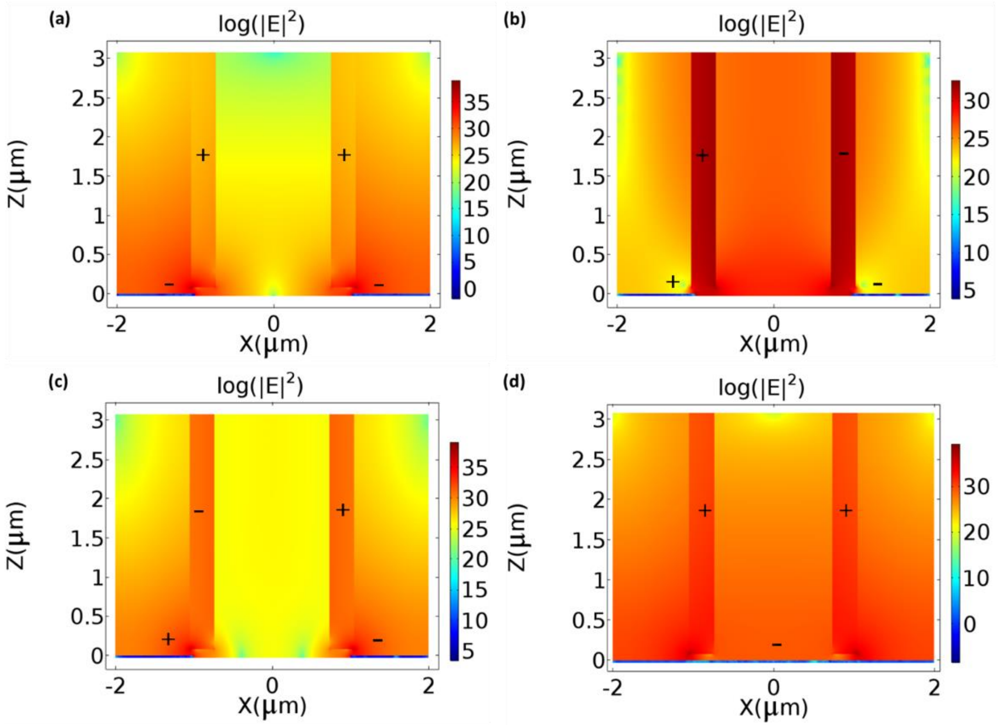

Fig. S1 COMSOL simulation **log** $(|\mathbf{E}|^2)$ of various phases of electrodes with the gap (**a**), (**b**), and (**c**) and without the gap (**d**). Only (**a**) case separate the maximum and the minimum at the edges and the center, respectively. Therefore, we apply the same phase of AC voltage of (**a**).

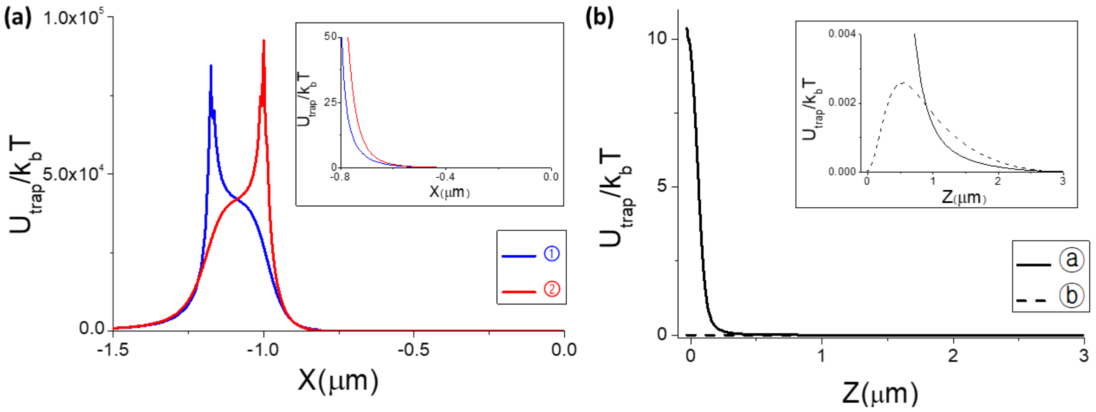

Fig. S2 Same simulation with Fig. 2(**c**),2(**d**) with the NDEP condition ($f_{CM} = -0.5$). (**a**) Force of the direction toward the center that is NDEP force. (**b**) shows particles under NDEP near electrodes go upward by NDEP force, but DEP energy is not bigger than Brownian motion energy near the center.

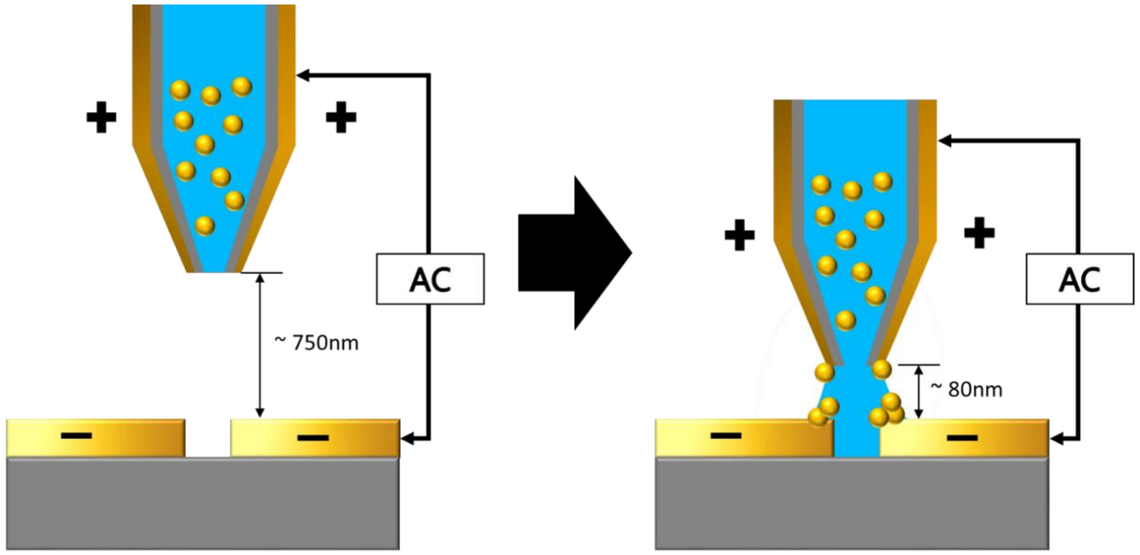

Fig. S3 Experimental process of DEP. AC voltage is applied while approaching before the formation of a water channel when the distance between the orifice and the substrate is almost 750 nm.

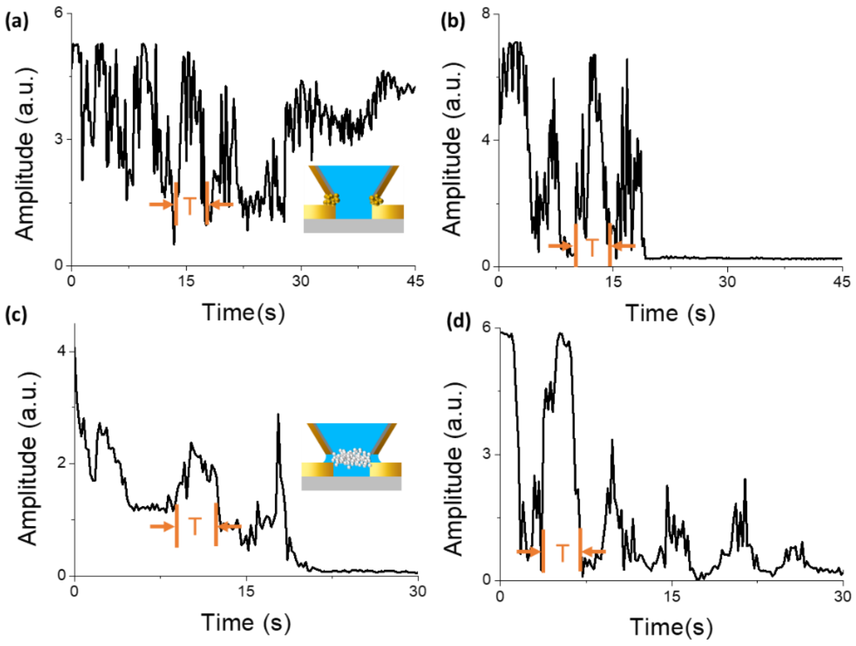

Fig. S4 Amplitude signals of QTF when applying AC voltage onto the quadrupole electrodes after liquid nanochannel was formed. (**a**) Au nanoparticles, PDEP. (**b**) Silica nanoparticles, PDEP. (**c**) Silica nanoparticles, NDEP. (**d**) Silica and au nanoparticles (selective deposit). In all cases, 3~4 oscillations of the same period (~5 s) occurred. After those oscillations, a liquid channel gradually vanished in (**a**) Au case, because aggregated Au nanoparticles increase current and generated thermal energy vaporize the meniscus. However, amplitude signals in (**b**), (**c**) silica case show solidification behavior caused by connecting electrodes by the aggregated silica nanoparticles such as the inset image in (**c**). (**d**) mixed case also shows solidification behavior, but it is not as fast as (**b**), (**c**) because of the gold nanoparticles.

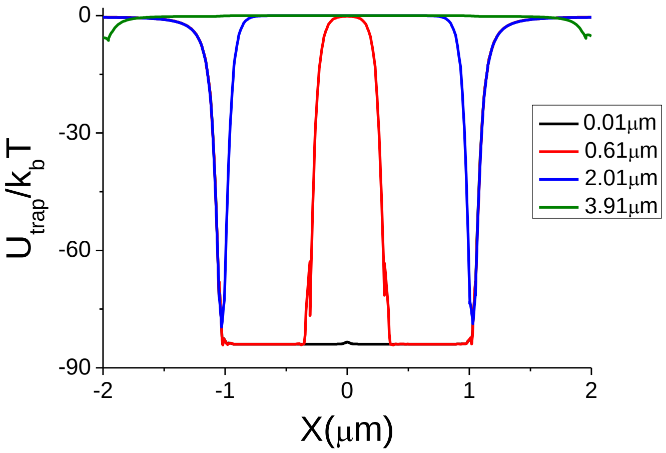

Fig. S5 COMSOL simulation of trap energy depending on the width of the gap. In this simulation, the diameter of the pipette is 2.1 μm, and $\boldsymbol{V_{peak}}$ = 1.0 V, 15 MHz was used. When the width of the gap is 0.01 μm or 3.91 μm, the gradient of trap energy does not exist inside the orifice of the pipette, so DEP force does not affect. Also, when the width of the gap is 0.61 μm, PDEP spot (potential minimums) is much wider than the NDEP spot (a potential maximum at the center), so the DEP effect does not work properly as shown in Fig. S6(b). Therefore, the ratio of the diameter of the pipette and the gap distance should be near 1.

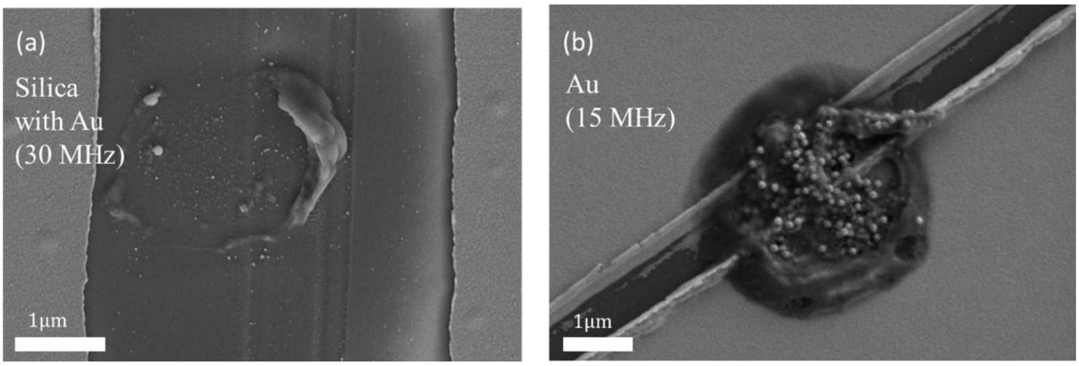

Fig. S6 SEM images of inappropriate ratio results. Both images do not show the expected result. (**a**) A smaller pipette case. 100 nm diameter silica nanoparticles and 20 nm diameter gold nanoparticles, 1 μm diameter pipette and 2 μm gap, $\boldsymbol{V}_{\mathbf{peak}}$ = 2.5 V, and 30 MHz frequency are used. (**b**) A smaller gap case. 50 nm diameter gold nanoparticles, ~ 1.5 μm diameter pipette, and ~ 0.35 μm gap, $\boldsymbol{V}_{\mathbf{peak}}$ = 1.0 V and 15 MHz frequency are used.

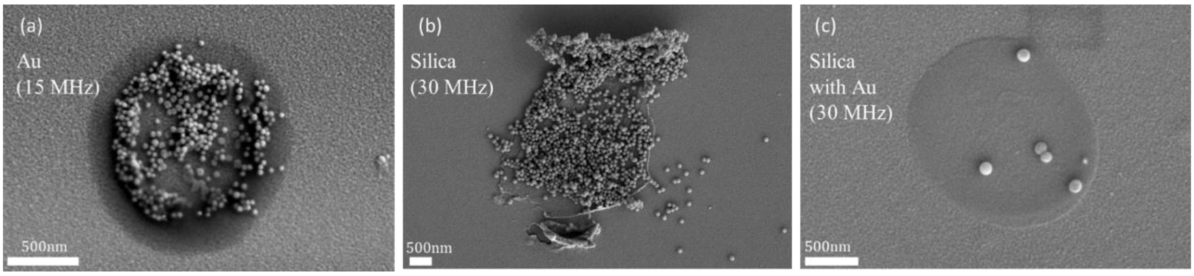

Fig. S7 SEM images of no gap cases. Three images do not show a particular regularity. (**a**) An au nanoparticle solution case. 50 nm diameter gold nanoparticles, 1.5 μm diameter pipette, $\boldsymbol{V}_{\mathbf{peak}}$ = 1.0 V and 15 MHz frequency are used. (**b**) A silica nanoparticle solution case. 100 nm diameter silica nanoparticles, 3 μm diameter pipette, $\boldsymbol{V}_{\mathbf{peak}}$ = 2.5 V and 30 MHz frequency are used. (**c**) A mixed solution case. 100 nm diameter silica nanoparticles and 20 nm diameter gold nanoparticles, 1.8 μm diameter pipette, $\boldsymbol{V}_{\mathbf{peak}}$ = 2.5 V and 30 MHz frequency are used.

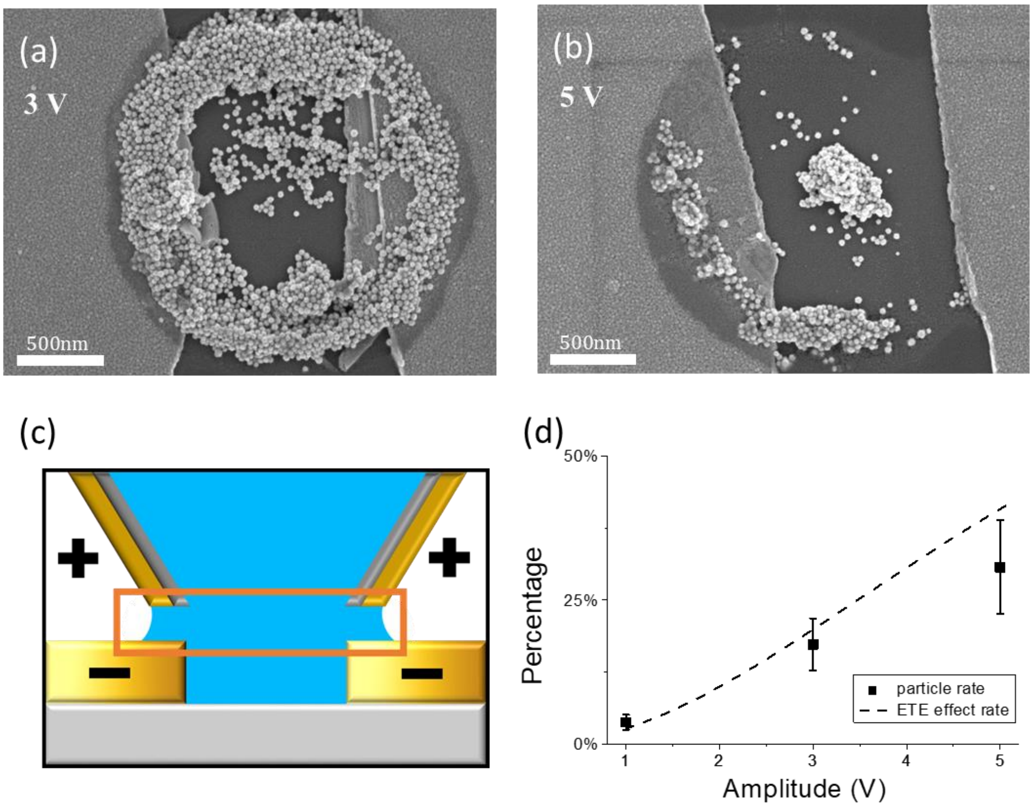

Fig. S8 AC Amplitude dependence of PDEP effect of Au nanoparticles. (**a, b**) SEM images of PDEP experiment with the different amplitude of AC voltage. (**a**) $\boldsymbol{V}_{\mathbf{peak}}$ = 3.0 V, 15 MHz frequency (**b**) $\boldsymbol{V}_{\mathbf{peak}}$ = 5.0 V 15 MHz frequency (**c**) Schematic diagram of simulation region of electrothermal effect. We assumed that electrical thermal loss goes to the water in the orange square region. (**d**) Graph of the particle rate (black squares) and the ETE effect rate (a dashed line) versus amplitude. The particle rate means the number rate of not appropriately deposited particles and the ETE effect rate means the theoretical calculation of the ETE effect versus the DEP effect rate. Typically, it is expected that Higher amplitude makes higher spatial deposit accuracy because of stronger DEP force. However, the opposite results are produced because of the ETE effect.
